# Nationwide Survey of Work Environment, Work-Life Balance and Burnout among Psychiatrists in Japan

**DOI:** 10.1371/journal.pone.0055189

**Published:** 2013-02-13

**Authors:** Wakako Umene-Nakano, Takahiro A. Kato, Saya Kikuchi, Masaru Tateno, Daisuke Fujisawa, Tsutomu Hoshuyama, Jun Nakamura

**Affiliations:** 1 Department of Psychiatry, University of Occupational and Environmental Health, Kitakyushu, Japan; 2 Department of Neuropsychiatry, Graduate School of Medical Sciences, Kyushu University, Fukuoka, Japan; 3 Innovation Center for Medical Redox Navigation, Kyushu University, Fukuoka, Japan; 4 Department of Psychiatry, Tohoku University, Graduate School of Medicine, Sendai, Japan; 5 Department of Neuropsychiatry, Sapporo Medical University, Sapporo, Japan; 6 Psycho-Oncology Division, Research Center for Innovative Oncology, National Cancer Center Hospital East, Kashiwa, Japan; 7 Department of Environmental Epidemiology, University of Occupational and Environmental Health, Kitakyushu, Japan; 8 Ushibuka City Hospital, Ushibuka, Amakusa, Japan; Chiba University Center for Forensic Mental Health, Japan

## Abstract

**Background:**

Psychiatry has been consistently shown to be a profession characterised by ‘high-burnout’; however, no nationwide surveys on this topic have been conducted in Japan.

**Aims:**

The objective of this study was to estimate the prevalence of burnout and to ascertain the relationship between work environment satisfaction, work-life balance satisfaction and burnout among psychiatrists working in medical schools in Japan.

**Method:**

We mailed anonymous questionnaires to all 80 psychiatry departments in medical schools throughout Japan. Work-life satisfaction, work-environment satisfaction and social support assessments, as well as the Maslach Burnout Inventory (MBI), were used.

**Results:**

Sixty psychiatric departments (75.0%) responded, and 704 psychiatrists provided answers to the assessments and MBI. Half of the respondents (n = 311, 46.0%) experienced difficulty with their work-life balance. Based on the responses to the MBI, 21.0% of the respondents had a high level of emotional exhaustion, 12.0% had a high level of depersonalisation, and 72.0% had a low level of personal accomplishment. Receiving little support, experiencing difficulty with work-life balance, and having less work-environment satisfaction were significantly associated with higher emotional exhaustion. A higher number of nights worked per month was significantly associated with higher depersonalisation.

**Conclusions:**

A low level of personal accomplishment was quite prevalent among Japanese psychiatrists compared with the results of previous studies. Poor work-life balance was related to burnout, and social support was noted to mitigate the impact of burnout.

## Introduction

Burnout is the feeling of physical, emotional, and mental exhaustion that is caused by long-term involvement in situations that are emotionally damaging [1]. Professionals caring for people with long-term and serious illnesses are frequently exposed to distressing emotional situations and profound suffering, which can lead to burnout. Maslach et al. [2] described burnout as the point at which important, meaningful, and challenging work becomes unpleasant, unfulfilling, and meaningless. Indeed, at this point, energy turns into exhaustion, involvement leads to cynicism, and efficacy is replaced by ineffectiveness. Psychiatry has been consistently shown to be a profession characterised by signs of high burnout [1]. Indeed, psychiatrists are at higher risk for mental illness, burnout and suicide compared with other health professionals [3]–[5]. Deahl and Turner [6] identified violence and the fear of violence, limited resources, crowded inpatient wards and an increasing culture of blame creeping into mental health services as the main sources of stress for psychiatrists. A large survey of psychiatrists identified working long duty hours, dealing with the difficult and hostile relatives of patients, arranging admissions, managing paper work, balancing personal and professional lives and managing suicidal or homicidal patients as particularly stressful experiences [1]. Lasalvia et al. reported that psychiatrists have the highest levels of burnout among mental health staff, and burnout was mostly predicted by a higher frequency of face to face interaction with users, longer tenure in mental healthcare, weak work group cohesion and perceived unfairness [7]. Personal and organisational factors have been associated with stress and burnout [8], whereas job satisfaction was found to have a protective effect against stress and burnout [9].

Balancing one's private and professional life (i.e., work-life balance) is also important for healthcare professionals. Work-life conflict is defined as the conflict between work and family demands, as well as the conflict between work and other role expectations and responsibilities in one's private life [10]. Work-life conflict is reported to be a major contributing factor to work stress for those working in the health-care sector in many industrialised countries. Over the past few decades, increased work demands, working hours, shift work and staff shortages have been associated with an imbalance between work and personal life [11]–[12]. Work-life conflict is a recognised risk factor for work stress and burnout; however, to date, the relationship between work-life conflict and work stress or burnout has not been investigated [13].

There are 80 medical schools in Japan. Medical schools have clinical, research and educational roles in society. However, the number of doctors at medical schools has decreased dramatically since the introduction of a new 2-year postgraduate medical education system in 2004 [14]–[16]. Medical faculties are overloaded with clinical duties and educational responsibilities due to the shortage of medical doctors on medical school faculties. As a result, medical faculty have no additional time for research. It has recently been suggested that the number of young scientists has decreased in Japan [17]. This trend has been observed in all medical specialties in Japan. In psychiatry departments across Japan, there has been a notable shortage of medical school faculty, as well as a decline in the number of postgraduate psychiatry students [18]–[20].

To date, no studies have been conducted in Japan to investigate the relationship between work environment, work-life balance and burnout among psychiatrists working in medical schools. Therefore, the current study was developed to survey burnout, work environment, and work-life balance in Japan. The aims of our study were as follows: to estimate the prevalence of burnout among Japanese psychiatrists; to estimate job satisfaction among Japanese psychiatrists; and to ascertain the relationship between socio-demographic variables, work environment satisfaction, work-life balance satisfaction and burnout among psychiatrists working in medical schools in Japan.

## Methods

### Ethics Statement

The protocol of this study was approved by the Ethics Committee at the University of Occupational and Environmental Health. All respondents participated in this study without any incentive. Similarly, all authors and subjects involved in this study declared themselves free of any conflict of interest relating to the study.

### Respondents and Procedure

We mailed a questionnaire to all 80 psychiatric departments in Japan between March and May 2011. Questionnaires were distributed to psychiatrists in each department. The subjects were informed that participation in the study was voluntary and that their responses would be anonymous.

### Questionnaire

Questionnaires were anonymous and self-administered. They gathered information on socio-demographics, work-life balance satisfaction, work environment satisfaction, burnout, and social support.

#### Burnout

The Maslach Burnout Inventory (MBI) [21] is a self-administered questionnaire consisting of 22 items rated on a 7-point Likert scale (possible range, 0–6) that measures three subscales of burnout syndrome, namely emotional exhaustion (9 items), depersonalisation (5 items), and personal accomplishment (8 items). Higher scores on emotional exhaustion and depersonalisation indicate higher levels of burnout, while lower scores on personal accomplishment indicate higher levels of burnout.

High levels of burnout are defined as a high level of emotional exhaustion (score range, 27 or higher), a high level of depersonalisation (score range, 10 or higher), and a low level of personal accomplishment (score range, 33 or lower), average levels of burnout are defined as an average level of emotional exhaustion (score range, 19–26), an average level of depersonalisation (score range, 6–9), and an average level of personal accomplishment (score range,34–39), and low levels of burnout are defined as a low level of emotional exhaustion (score range,18 or lower), a low level of depersonalisation (score range, 5 or lower), and an average level of personal accomplishment (score range, 40 or higher), based on normative data from a sample of American health professionals (physicians and nurses) [21]. Maslach et al. defined emotional exhaustion as the feeling of being emotionally over-extended and exhausted by one's work. Depersonalisation was defined as a negative, cynical attitude and having impersonal feelings toward clients, who are treated solely as objects. Finally, “reduced personal accomplishment” was defined as people feeling that they had low productivity and little achievement in their work. The higher the emotional exhaustion and depersonalisation and the lower the level of personal accomplishment, the more a physician will suffer from burnout, the primary symptom of which is believed to be emotional exhaustion. The reliability of the Japanese version of the MBI has been proven [22], and many Japanese researchers have used it in their research [23], [24].

#### Social support

The social support in the workplace questionnaire is a self-administered questionnaire consisting of 14 items rated on a 5-point Likert scale (possible range, 1–5) that measures two subscales of support: emotional support (8 items) and instrumental support (6 items). Higher scores on each of these subscales indicate greater amounts of support [25]. This questionnaire was developed to be suitable to the Japanese occupational situation. It has become standard in Japanese occupational mental health and has been used widely [26].

#### Work-life balance satisfaction

We prepared the following question: “Are you experiencing difficulty with your work-life balance?”, and answers were given on a Likert scale ranging from 1 to 5, with a higher score indicating a greater feeling of difficulty with work-life balance.

#### Work environment satisfaction

We prepared the following question: “Are you satisfied with your work environment?”, and answers were given on a Likert scale ranging from 1 to 5, with a higher score indicating greater work environment satisfaction.

### Statistical Analysis

Chi-squared tests were conducted to assess burnout rates. Spearman's correlations were calculated between work-life balance or work environment satisfaction and burnout subscales. We performed multivariate linear regression to test the associations between individual factors and total scores on the MBI. Variance inflation factor (VIF) was used to check for multicollinearity. VIF is used in the analysis of multivariate data as a method of determining when estimates of the statistical coefficients in the model are unstable. In general the largest VIF of all the markers in the test is used to indicate the stability of the test and values above 2 are judged to indicate an unstable model [27]. All of the statistical analyses were conducted using SAS, version 9.1 (SAS Institute, Cary, North Carolina, USA). The level of significance for the results was set at p<0.05.

## Results

### Psychiatrist characteristics

Sixty psychiatric departments (75.0%) responded, and 704 psychiatrists completed the questionnaires. Forty-nine of the 60 psychiatric departments answered the number of the actual questionnaire which was distributed to. The response rate was 70.0% (349/499), and the majority of the respondents were male (77.4%). The baseline characteristics of the psychiatrists who responded are summarised in Table 1.

**Table 1:** Baseline characteristics of psychiatrists in the sample group.

	Total (n = 704) n (%) ^a^
Age (years)	
Mean ± S.D.	37.2±7.5
Gender	
Male	545 (77.4)
Female	159 (22.6)
Experience as a medical doctor (years)	11.1±7.7
Experience as a psychiatrist (years)	9.6±8.2
Position	
Professor or Associate Professor	151 (22.8)
Assistant Professor	237 (35.7)
Psychiatric Resident	171 (25.8)
Postgraduate Student	104 (15.7)
Marital status	
Married	462 (66.6)
Not married	232 (33.4)
Children status	
Have children	351 (50.7)
Do not have children	341 (49.3)
Average working hours per week	
Less than 40 hours	38 (5.5)
40 to less than 50 hours	148 (21.4)
50 hours or more	505 (73.1)
Number of nights worked per month	
None	83 (12.0)
Less than 4	245 (35.3)
5 to 9	282 (40.6)
10 or more	84 (12.1)

a Percentage of the total number of valid values for each variable.

### Rates of work-life balance and environmental satisfaction

**Figure 1:**
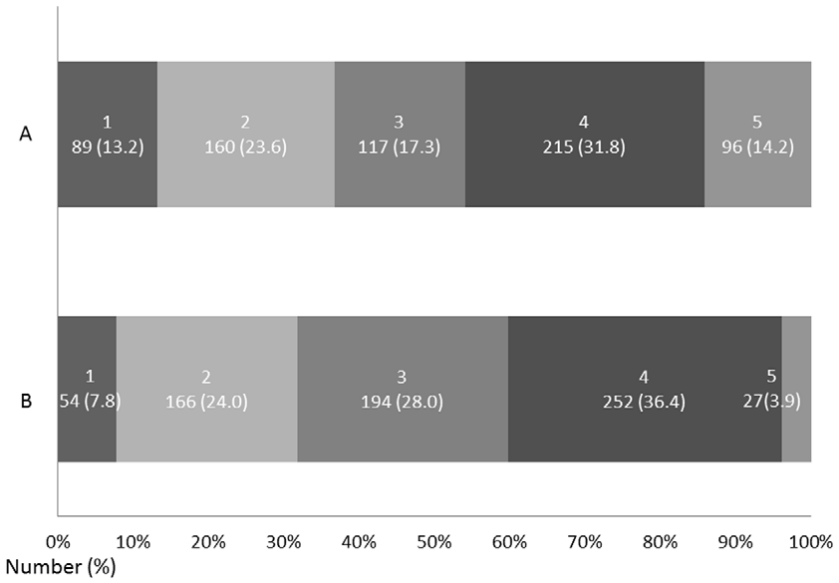
Rates of work-life balance and environmental satisfaction. A: Are you experiencing difficulties in maintaining your work-life balance? (1: not at all, 5: very much) B: Are you satisfied with your work environment? (1: not satisfied at all, 5: very much satisfied).

Figure 1 shows the rates of work-life balance and environment satisfaction. Slightly less than half of the respondents (n = 311, 46.0%) experienced difficulties in maintaining their work-life balance. Approximately one-third of the respondents (n = 220, 31.8%) were not satisfied with their work environment.

### Mean MBI scores and burnout rates

The mean MBI scores and burnout rates are shown in Table 2.

**Table 2:** Means MBI scores and burnout rates.

	Mean ± S.D.	Low burnout	Average burnout	High burnout	P ^a^
Emotional Exhaustion	18.9±11.4	396 (56.3)	160 (22.7)	148 (21.0)	<0.001
Depersonalisation	4.6±5.0	499 (70.9)	119 (16.9)	86 (12.2)	<0.001
Personal Accomplishment	26.2±11.1	81 (11.5)	116 (16.5)	507 (72.0)	<0.001

a Chi-squared test; the results of three burnout subsets were compared.

The mean emotional exhaustion score provided evidence of average burn out, the mean depersonalisation score provided evidence of low burnout, and the mean personal accomplishment score provided evidence of high burnout. The majority of psychiatrists (n = 507, 72%) expressed low levels of personal accomplishment, i.e., high burnout level.

### Association between work-life balance satisfaction and burnout

**Figure 2:**
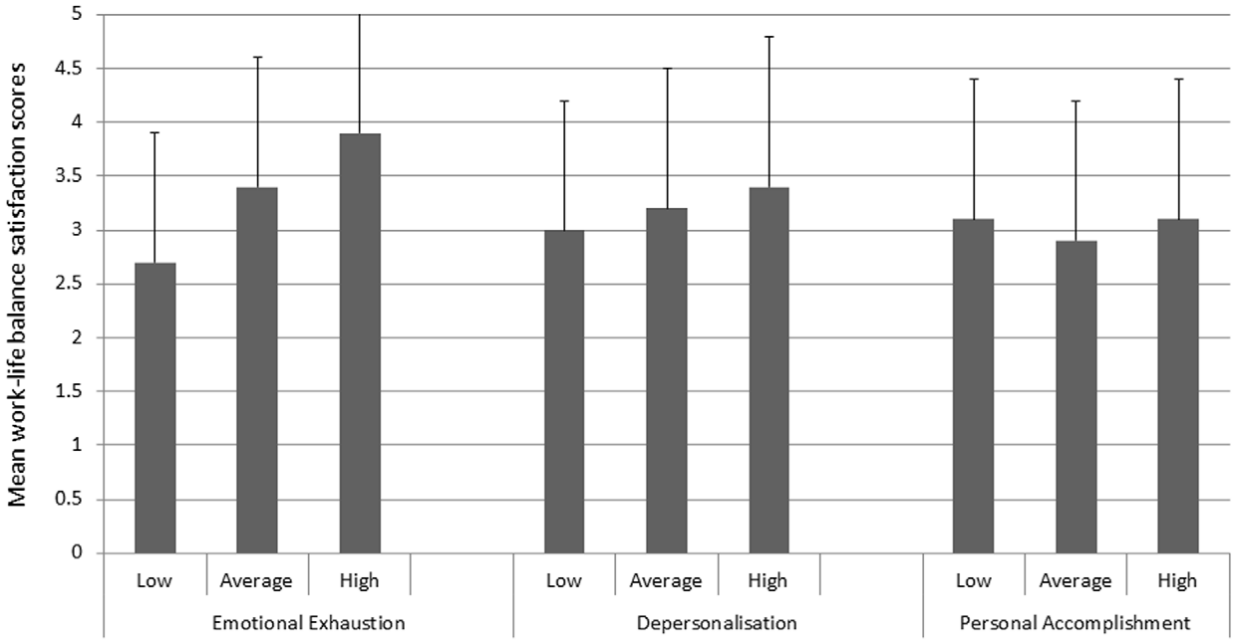
Association between work-life balance satisfaction and burnout. Are you experiencing difficulties in maintaining your work-life balance? (1: not at all, 5: very much).

There were significant correlations between average work-life balance satisfaction scores and burnout scores. Experiencing difficulties with work-life balance increased emotional exhaustion (r = 0.40350, p<0.0001) and depersonalisation (r = 0.09221, p = 0.177). There was no significant correlation between the mean work-life balance satisfaction scores and personal accomplishment (p = 0.2110). These trends are shown clearly in Figure 2.

### Association between work environmental satisfaction and burnout

**Figure 3:**
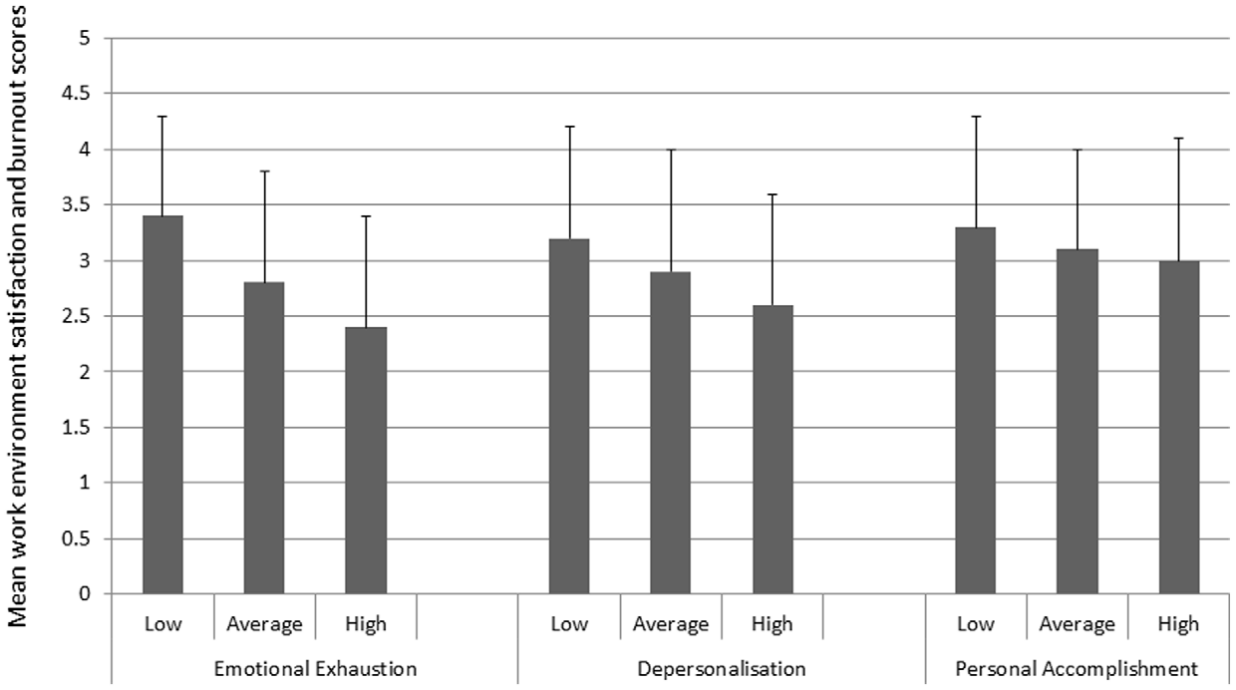
Association between work environmental satisfaction and burnout. Are you satisfied with your work environment? (1: not satisfied at all, 5: very much satisfied).

There were significant correlations between the mean work environment satisfaction and burnout scores. Satisfaction decreased with increased emotional exhaustion (r = −0.42680, p<0.0001), increased depersonalisation (r = −0.17765, p<0.0001) and decreased personal accomplishment (r = 0.11076, p = 0.0044). These trends are shown clearly in Figure 3.

### Factors associated with total MBI scores

The associations between individual factors and total MBI scores, as determined using a multivariate linear regression analysis, are shown in Table 3. If we had included the items “Age”, “Experience as a medical doctor (years)”, and “Experience as a psychiatrist (years)”, each VIF would have been over 2; that is why we selected only the 9 items in Table 3. The best performing model was that for emotional exhaustion (total adjusted R^2^ = 0.29). Receiving little social support (β = −0.22, p<0.0001), experiencing greater difficulties in maintaining work-life balance (β = 0.28, p<0.0001), and having low work environment satisfaction (β = −0.24, p<0.0001) were significantly associated with higher emotional exhaustion. Being male (β = −0.10, p = 0.0239), having a higher number of nights worked per month (β = 0.14, p = 0.0011), receiving little social support (β = −0.14, p = 0.0004), experiencing greater difficulties with work-life balance (β = 0.11, p = 0.0122), and having low work environment satisfaction (β = −0.10, p = 0.0174) were significantly associated with higher depersonalisation. Getting a promotion (β = −0.10, p = 0.0257) and receiving more social support (β = 0.10, p = 0.0138) were significantly associated with higher personal accomplishment scores.

**Table 3:** Factors associated with burnout and stress: multivariate linear regression analysis (n = 704).

	Emotional Exhaustion			Depersonalization			Personal Accomplishment		
	β	p	VIF	β	p	VIF	β	p	VIF
Gender a	−0.02	0.6068	1.31	−0.10	0.0239 *	1.30	0.02	0.5976	1.30
Position b	0.06	0.1364	1.32	0.05	0.2835	1.32	−0.10	0.0257 *	1.34
Marital status c	0.06	0.1892	1.77	−0.02	0.7568	1.76	−0.09	0.1052	1.78
Children status d	0.02	0.6250	1.85	0.02	0.6487	1.86	0.03	0.6102	1.86
Average working hours per week e	0.04	0.2540	1.26	−0.06	0.1573	1.24	0.02	0.6114	1.25
Number of nights worked per month f	0.02	0.6461	1.36	0.14	0.0011 *	1.35	−0.03	0.5361	1.37
Total scores of social support	−0.22	<. 0001 *	1.15	−0.14	0.0004 *	1.15	0.10	0.0138 *	1.14
Difficulties experienced with work-life balance g	0.28	<. 0001 *	1.28	0.11	0.0122 *	1.27	−0.03	0.5546	1.26
Work environment satisfaction g	−0.24	<. 0001 *	1.34	−0.10	0.0174 *	1.34	0.08	0.0799	1.32
Total R^2^	0.30			0.10			0.04		
Total adjusted R^2^	0.29			0.09			0.03		

β: standardised regression coefficient.

VIF: variance inflation factor.

a Coded as: 0 =  Male; 1 =  Female.

b Coded as: 1 =  Professor or Associate Professor; 2 =  Assistant Professor; 3 =  Psychiatric Resident; 4 =  Postgraduate Student.

c Coded as: 0 =  Married; 1 =  Not married.

d Coded as: 0 =  Have children; 1 =  Do not have children.

e Coded as: 1 =  Less than 40 hours; 2 = 40 to less than 50 hours; 3 = 50 hours or more.

f Coded as: 1 =  None; 2 =  Less than 4 times; 3 = 5 to 9 times; 4 = 10 times or more.

g Possible range: 1–5. Higher scores indicate higher satisfaction levels.

* Statistically significant variables.

## Discussion

This is the first nationwide survey conducted to determine the relationship between work environment, work-life balance and burnout among psychiatrists working in medical schools in Japan. The present study demonstrated several findings. First, 21.0% of the respondents had a high level of emotional exhaustion, 12.0% had a high level of depersonalisation, and 72.0% had a low level of personal accomplishment. Second, 46.0% of responders experienced difficulties in maintaining their work-life balance, and 31.8% were not satisfied with their work environment. Experiencing difficulties maintaining a work-life balance and having decreased work environment satisfaction were significantly related to burnout subscales. Finally, having adequate social support was the factor most strongly associated with lower levels of burnout.

It has been reported that levels of emotional exhaustion among mental health staff are significantly higher than in the normative group [7] [28]. In previous studies examining burnout in psychiatrists [29]–[30], high levels of emotional exhaustion (33 and 49%), high levels of depersonalisation (13 and 39%) and low levels of personal accomplishment (34 and 22%) were observed. The present study revealed a lower prevalence of emotional exhaustion, a lower prevalence of depersonalisation and a higher level of decreased feelings of personal accomplishment compared with the results of previous studies conducted in psychiatrists. Comparing MBI among other occupations, in a recent meta-analysis [31] that looked at 45 studies that explored the factorial structure of the MBI there were no Japanese samples. Fujiwara et al [32] have reported on MBI among home care workers in Japan. In their study, high levels of emotional exhaustion (24.9%), high levels of depersonalisation (5.1%) and low levels of personal accomplishment (28.2%) were observed. The present study revealed a similar prevalence of emotional exhaustion, a higher prevalence of depersonalisation and a higher level of decreased feelings of personal accomplishment compared with the results of previous studies conducted in home care workers in Japan.

Factors associated with increased emotional exhaustion included being male and working 12-hour shifts [33], as well as clinical supervision and leadership style [34].

A higher number of nights worked per month was significantly associated with higher depersonalisation in this study. Spending over 50% of the work day in direct client contact, having short clinical experiences [35] and being involved in suboptimal patient care [36] were associated with increased depersonalisation. Psychiatrists who have short clinical experience, such as psychiatric residents who work many night shifts, may have influenced the relationship found in this study.

The age of subjects in this study is less than that in previous studies on psychiatrists. In this study, there was a higher rate (72.0%) of feelings of low levels of personal accomplishment. Factors associated with a lower sense of personal accomplishment were working in wards with difficult patients, being male, having a heavy workload [33], having a high percentage of patients who suffer from schizophrenia [37], and having a negative relationship with a senior psychiatrist [38]. In this study, getting a promotion (β = −0.10, p = 0.0257) and receiving more social support (β = 0.10, p = 0.0138) were significantly associated with higher personal accomplishment scores. We did not investigate what kind of patients the subjects had, but this study was conducted in a medical school setting, where one of the roles is to treat difficult patients (e.g., treatment-resistant patients). The therapy provided to patients in medical schools is administered by a team comprised of primary doctors, who have limited experience, and the superiors who train them. A higher level of personal accomplishment was associated with the perception that the decision process is democratic, rather than “top down”, and that decisions are based on awareness of a given situation, accurate information, and staff involvement in decisions that directly influence the staff's work [29].

Normally in medical schools in Japan, professors or associate professors who are in high positions work as superiors, and psychiatric residents who are in low positions work as primary doctors. This study's results of high rates of a low level of personal accomplishment and the relationship between personal accomplishment and position or social support might have been influenced by the many psychiatric residents who sometimes have difficult patients and do not have a very positive relationship with their superiors. Lsalvia et al. reported that improving the workplace atmosphere within psychiatric services should be one of the most important targets in staff burnout prevention strategies [7]. They also have reported that a poorly cohesive work group is at higher risk of burnout. Group cohesion relates directly to job satisfaction, namely through human relations with fellows and supervisors. The prevalence of burnout subsets in this study may have been influenced by the unique challenges faced by psychiatrists working in medical schools in Japan. It is also possible that Japanese national character influenced the high rate of burnout in this study, in which there was a high rate (72.0%) of feelings of low level of personal accomplishment. Asai et al [39] have reported a similar high rate (62%) of feelings of low levels of personal accomplishment in a study of MBI among physicians engaged in end-of life care for cancer in patients in Japan. In a previous study [40] investigating the factor structure of MBI among nurses in 8 countries (Japan, USA, Canada, UK, Germany, New Zealand, Russia and Armenia), only in Japan was there a very weak or almost no relationship between personal accomplishment and feelings of emotional exhaustion and depersonalization. In Asian countries, professionals may have unique ways of defining their accomplishment at work [41]. Future research is needed to understand how psychiatrists in Japan rate their work performance and accomplishment, and what are the factors associated with their accomplishment at work.

Negative spill-over effects and role conflicts between work and family or one's private life indicating a work-life imbalance have also repeatedly been shown to be strongly associated with job stress and general psychological or emotional stress [42]–[43] and/or burnout symptoms [10], [42] [44]. In this study, experiencing difficulties in achieving a work-life balance was significantly and positively correlated with increased emotional exhaustion and depersonalisation (Figure 2), as well as factor associated with high level of emotional exhaustion and depersonalisation (Table 3). Garfinkel et al. [45] reported that paying attention to one's non-professional life has a protective role against burnout. These authors found that the best predictor of personal satisfaction was overall satisfaction with non-professional aspects of one's life. Keeton et al. [46] reported that burnout is an important predictor of career satisfaction, and control over one's schedule and work hours are the most important predictors of work-life balance and burnout among physicians.

In this study, social support was the factor most strongly associated with low burnout rates. Coping mechanisms that have been adopted to effectively deal with this stress include receiving support from one's partner, participating in recreational activities and maintaining good relationships with colleagues [47]. Dallender et al. [48] reported that the most important coping mechanisms for psychiatrists are the support of a loved one and one's colleagues. This strategy was used by psychiatrists to help them deal with a patient's suicide [49]. Receiving support for work-related problems was a key factor in reducing work-related exhaustion and increasing mental energy in psychiatrists.

The issue of the shortage of medical faculty has been reported not only in Japan but also in the USA. Recent national survey data from the American Association of Medical Colleges reported a staggering 38–40% attrition rate of academic medical faculty over a ten year period [50]. Bunton et al [51] have reported the predictors of workplace satisfaction for medical school faculty among large samples in the USA. Respondents were mostly satisfied on global satisfaction measures including satisfaction with their department (71.3%) and medical school (63.5%) and whether they would again choose to work at their medical school (70.2%). Predictors across models include organization, governance, and transparency; focus of mission; recruitment and retention effectiveness; department relationships; workplace culture; and nature of work. Because the clinical track is structurally different from the instructional track, Chung et al [52] conducted a survey to identify and contrast predictors of job satisfaction between the two tracks among a medical school faculty in the USA. They reported that the significant predictors of job satisfaction for both tracks included areas of autonomy, meeting career expectations, work-life balance, and departmental leadership. Unique to the clinical track, compensation and career advancement variables also emerged as significant predictors. In the present study, 68.2% were satisfied with their work environment, which is similar to a previous study among medical faculty in the USA. As we did not investigate predictors of job satisfaction directly, further studies are needed in Japan.

This study has some limitations. First, a cross sectional design can't reveal causality in this study. We did not compare data from a random sample representing a wide variety of occupations from among the general population, or a random sample of psychiatrists who work in general hospitals other than medical schools. Second, although the response rate of 75.0% was high, the data are not reflective of all psychiatrists who work in medical schools. According to the latest data provided by the Japanese Ministry of Health, Labour and Welfare, the total number of psychiatrists in Japan is 14,201, accounting for 5.06% of all medical doctors in Japan in 2010 (on-line database of JMHLW; http://www.mhlw.go.jp/toukei/youran/indexyk_2_2.html). The number of medical doctors who worked in medical schools was 48,557 in 2010. Given this data, we estimated the number of psychiatrists who worked in medical schools to be 2,456. Thus, the subjects in this study account for 28.7% of all psychiatrists who work in medical schools. Third, the classification of burnout levels was based on the data of American health professionals, thus this might not be true of Japanese psychiatrists. Forth, the validity of the questionnaire measuring social support in this study might not have been established yet, and we did not use validated scales to assess work-life balance and job satisfaction. Fifth, we did not investigate important background information such as subspecialty of psychiatry, work expertise, working units (inpatients or outpatients), financial variables and leisure hours among subjects. Sixth, the model did not explain “Depersonalization” or “Personal Accomplishment” very well because the total adjusted R^2^ was low. Finally, there may be a possible response bias, as many burned out or dissatisfied psychiatrists may not have responded. Indeed, their non-responsiveness could have been influenced by burnout or job dissatisfaction.

In conclusion, there was a high prevalence of low levels of personal accomplishment among Japanese psychiatrists who work in medical schools compared with previous studies. Work-life balance was associated with burnout, and emotional support was important in mitigating burnout.
